# SARS-CoV-2 infection in nonhuman primates alters the composition and functional activity of the gut microbiota

**DOI:** 10.1080/19490976.2021.1893113

**Published:** 2021-03-08

**Authors:** Harry Sokol, Vanessa Contreras, Pauline Maisonnasse, Aurore Desmons, Benoit Delache, Valentin Sencio, Arnaud Machelart, Angela Brisebarre, Lydie Humbert, Lucie Deryuter, Emilie Gauliard, Severine Heumel, Dominique Rainteau, Nathalie Dereuddre-Bosquet, Elisabeth Menu, Raphael Ho Tsong Fang, Antonin Lamaziere, Loic Brot, Celine Wahl, Cyriane Oeuvray, Nathalie Rolhion, Sylvie Van Der Werf, Stéphanie Ferreira, Roger Le Grand, François Trottein

**Affiliations:** a Sorbonne Université, INSERM, Centre De Recherche Saint-Antoine, CRSA, AP-HP, Saint Antoine Hospital, Gastroenterology Department, Paris, France; b INRAE, UMR1319 Micalis & AgroParisTech, Jouy En Josas, France; c Paris Center for Microbiome Medicine, Fédération Hospitalo-Universitaire, Paris, France; d Université Paris-Saclay, INSERM, CEA, Center for Immunology of Viral, Auto-immune, Hematological and Bacterial Diseases (Infectious Diseases Models for Innovative therapies/IDMIT), Paris, France; e Univ. Lille, US 41 - UMS 2014 - PLBS, U1019 - UMR 9017 - CIIL - Centre d’Infection Et d’Immunité De Lille, Lille, France; f Centre National De La Recherche Scientifique, Lille, France; g Institut National De La Santé Et De La Recherche Médicale U1019, Lille, France; h Centre Hospitalier Universitaire De Lille, Lille, France; i Institut Pasteur De Lille, Lille, France; j Centre National De Référence Virus Des Infections Respiratoires, Unité De Génétique Moléculaire Des Virus À ARN, GMVR, F75015, Institut Pasteur, UMR CNRS 3569, Université De Paris, Paris, France; k Genoscreen, Lille, France

**Keywords:** Gut microbiota, SARS-CoV-2, nonhuman primates, gut dysbiosis, metabolic output

## Abstract

The current pandemic of coronavirus disease (COVID) 2019 constitutes a global public health issue. Regarding the emerging importance of the gut-lung axis in viral respiratory infections, analysis of the gut microbiota’s composition and functional activity during a severe acute respiratory syndrome coronavirus 2 (SARS-CoV-2) infection might be instrumental in understanding and controling COVID 19. We used a nonhuman primate model (the macaque), that recapitulates mild COVID-19 symptoms, to analyze the effects of a SARS-CoV-2 infection on dynamic changes of the gut microbiota. 16S rRNA gene profiling and analysis of β diversity indicated significant changes in the composition of the gut microbiota with a peak at 10–13 days post-infection (dpi). Analysis of bacterial abundance correlation networks confirmed disruption of the bacterial community at 10–13 dpi. Some alterations in microbiota persisted after the resolution of the infection until day 26. Some changes in the relative bacterial taxon abundance associated with infectious parameters. Interestingly, the relative abundance of *Acinetobacter* (Proteobacteria) and some genera of the Ruminococcaceae family (Firmicutes) was positively correlated with the presence of SARS-CoV-2 in the upper respiratory tract. Targeted quantitative metabolomics indicated a drop in short-chain fatty acids (SCFAs) and changes in several bile acids and tryptophan metabolites in infected animals. The relative abundance of several taxa known to be SCFA producers (mostly from the Ruminococcaceae family) was negatively correlated with systemic inflammatory markers while the opposite correlation was seen with several members of the genus *Streptococcus*. Collectively, SARS-CoV-2 infection in a nonhuman primate is associated with changes in the gut microbiota’s composition and functional activity.

## Introduction

Coronavirus disease 2019 (COVID-19) is caused by severe acute respiratory syndrome coronavirus 2 (SARS-CoV-2), a novel coronavirus sharing significant sequence homology with SARS-CoV-1 and Middle East respiratory syndrome coronavirus.^1^ The majority of patients with COVID-19 present no symptoms or only mild symptoms (including fever, cough, and fatigue). In contrast, some patients with COVID-19 experience more severe symptoms (such as dyspnea), rapid deterioration, and the onset of serious complications such as acute respiratory distress syndrome (ARDS) and even multiple organ failure.^2–4^ The severity of COVID-19 is known to depend on the presence or absence of a range of comorbidities, including obesity, diabetes, hypertension, and advanced age^5–9^ and on inborn errors of immunity.^10,11^ Between 15 and 25% of patients with COVID-19 report gastrointestinal symptoms ranging from abdominal discomfort and loss of appetite to diarrhea, nausea and vomiting.^12,13^ Importantly, infectious viruses can be detected in fecal samples of COVID-19 patients suggesting that the digestive tract might be a site of viral replication and activity.^14–16^

The gastrointestinal tract hosts a highly diverse and dynamic microbial ecosystem, composed mostly of anaerobic bacteria, that is commonly referred to as the gut microbiota.^17^ The tightly regulated microbiota-host interplay influences many physiological functions such as digestion, metabolism, mucosal barrier integrity, organ functions and immune homeostasis. A body of preclinical and clinical evidence shows that the composition of the set of microorganisms that inhabit the gut is transiently altered in the context of an acute viral respiratory infection such as influenza.^18–26^ The picture emerging from these studies is that the absolute frequencies of potentially beneficial and opportunistic bacteria, respectively, decrease and increase during infection. Different mechanisms may explain these alterations in the gut microbiota’s composition, including the inflammatory process itself, loss of appetite (i.e., a lower fiber intake), hypoxia, and changes in local (epithelial) metabolism and immune function.^19,22–24^ The disruption of the microbial ecosystem modulates the clinical outcomes in several noncommunicable diseases, including metabolic disorders, cancer, and gastrointestinal diseases.^27–29^ In the context of viral respiratory infections, very few studies have focused on the potential secondary consequences of infection-associated changes in the gut microbiota. We and others have shown that gut microbiota collected from influenza-infected mice can transfer susceptibility to bacterial infection locally (*Salmonella Typhimurium*) and remotely (*Streptococcus pneumoniae*) – demonstrating the gut microbiota’s role in secondary disease outcomes.^19,22^ This question is highly relevant for COVID-19 because SARS-CoV-2 is found in the intestine in up to 25% of COVID-19 patients, and the microbiota alterations associated with pulmonary and intestinal insults are likely to influence the disease severity (for reviews, see^12,30^).

Changes in the human gut microbiota’s composition and function during a SARS-CoV or MERS-CoV infection have not previously been analyzed. However, several studies have reported gut microbiota alterations in patients with COVID-19^20,31–35^ . Of interest, SARS-CoV-2 infection is associated with a lower relative abundance of butyrate producers, such as several genera from the families Ruminococcaceae and Lachnospiraceae.^20^ Other bacterial species with known immunomodulatory potential such as *Faecalibacterium prausnitzii, Eubacterium rectale*, and bifidobacteria were also underrepresentend in COVID-19 patients^35^ . In contrast, the infection was associated with a significantly higher relative abundance of opportunistic bacteria, including *Streptococcus* (from the class Bacilli), *Rothia, Actinomyces, Ruminococcus* (*gnavus* and *torques*), and *Bacteroides* (*dorei* and *vulgatus*).^20,35^ Enrichment of these bacteria might lead to local and systemic inflammation^20,30,35^ . Furthermore, a study based on a shotgun metagenomics approach revealed the presence of opportunistic bacteria (including *Collinsella aerofaciens, Streptococcus infantis*, and *Morganella morganii*) in fecal samples of COVID-19 patients with a signature of high SARS-CoV-2 infectivity.^34^ In contrast, bacteria able to produce short-chain fatty acids (SCFAs) and tryptophan were enriched in fecal samples with a low-to-none SARS-CoV-2 infectivity signature.^34^ Interestingly, the samples with a high SARS-CoV-2 infectivity signature exhibited a higher microbiome functional capacity for *de novo* nucleotide biosynthesis, amino acid biosynthesis, and glycolysis.^34^ This alteration in bacterial functionality might be a consequence of SARS-CoV-2 infectivity in the gut or a disease severity factor.

In the context of an infection, human data are highly important but do not enable the entire course of infection (i.e. from before contamination until after resolution) to be monitored. We and others have shown that the nonhuman primate system is a relevant model for COVID-19.^36–40^ This system recapitulates the moderate disease that has been observed in the majority of human cases of COVID-19. In macaques, SARS-CoV-2 infection led to virus replication in the upper and lower respiratory tracts and animals experience lung pathology and respiratory disease, without overt clinical symptoms. In the present study, we took advantage of this model by monitoring changes over the course of infection in the gut microbiota’s composition and metabolic output. To this end, two closely related macaque species, namely Chinese rhesus macaques and Mauritian cynomolgus macaques were infected with SARS-CoV-2. Our results show that an experimental SARS-CoV-2 infection promotes an alteration in the gut microbiota in terms of taxonomic composition and, more importantly, functional activity. Some alterations in gut microbiota persisted after the resolution of the infection and so might have a role in the long COVID-19 symptoms that are currently being reported in humans.

## Results

### SARS-CoV-2 infection of rhesus macaques and cynomolgus macaques

**Figure 1.**
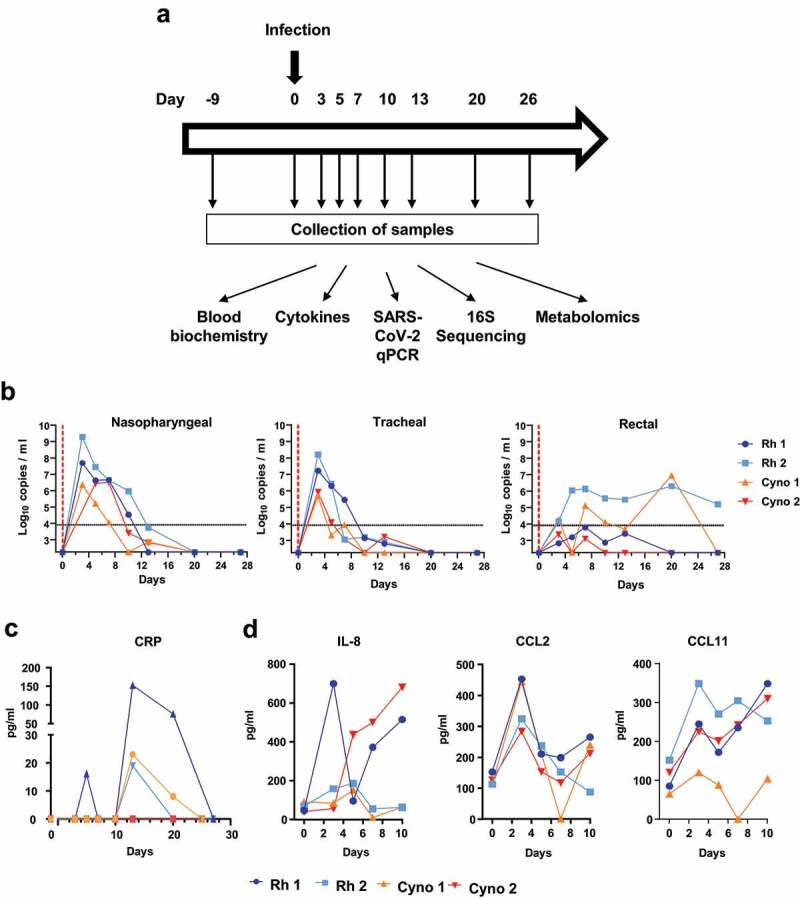
**A**, Infection of rhesus (Rh) macaques and cynomolgus (Cyno) macaques by SARS-CoV-2, and sample collection. Viral loads and systemic factors were quantified before infection (day −9 represents the basal line) and at different time points post-infection. Feces samples, but not other samples, were collected the day of infection (day 0). **B**, Viral loads (measured in an RT-qPCR assay) in nasopharyngeal swabs, tracheal swabs, and rectal swabs. The estimated limit of detection was 2.3 log10 copies of SARS-CoV-2 RNA per ml, and the estimated limit of quantification was 3.9 log10 copies per ml (dotted horizontal line); **C**, The plasma CRP concentration. **D**, Cytokine and chemokine concentrations in plasma. **B-D**, The time course analysis for each animal (n = 4). Basal line was adjusted to day 0 on the graphs

Rhesus macaques and cynomolgus macaques were infected intranasally and intratracheally with 2 × 10^7^ plaque-forming units (PFUs) of the clinical SARS-CoV-2 isolate hCoV-19/France/lDF0372/2020 (Figure 1a). Viral loads in nasopharyngeal and tracheal fluids were analyzed using an RT-qPCR assay. SARS-CoV-2 RNA was detected at 3 days post-infection (dpi); levels declined then rapidly, and the virus was undetectable at 20 dpi (Figure 1b). It is noteworthy that the viral load was higher in rhesus macaques than in cynomolgus macaques. The SARS-CoV-2 RNA was detected in rectal fluids of two macaques (one from each species) out of four sampled at 7 dpi (Figure 1b). Overall, the SARS-CoV-2 RNA load was higher (by a factor of 100 to 1000) in airway samples than in intestinal samples. As reported in patients,^34^ the SARS-CoV-2 RNA load decreased less rapidly over time in rectal samples than in nasopharyngeal and tracheal swabs; it could still be detected in rectal samples at 20 dpi and, for one animal, at 26 dpi (end of the study). No quantifiable viral RNA was detected in plasma at any time point in the study. No overt clinical signs and no significant weight loss were observed (Supplementary Figure 1a). However, the two rhesus macaques had transient diarrhea at 4 dpi.

Patients experiencing COVID-19 have enhanced levels of various inflammatory markers in blood.^4,5^ We observed elevated plasma levels of the inflammatory marker C-reactive protein (CRP, peaking at 13 dpi) and creatine kinase (peaking at 20 dpi) (three out of four animals) (Figure 1c and Supplementary Figure 1b). Analysis of plasma cytokine and chemokine levels revealed increased concentrations of interleukin (IL)-8, IL-1RA, C-C motif chemokine ligand (CCL)2, CCL11, and chemokine (C-X-C motif) ligand (CXCL)13 (Figure 1d and Supplementary Figure 1b). The time to peak for the various cytokines and chemokines and the duration of elevation varied from one animal to another. Collectively, our results are in line with the literature data^36–40^ showing that rhesus macaques and cynomolgus macaques are susceptible to SARS-CoV-2 experimental infection and develop systemic inflammation concomitantly to the early phase of viral replication in respiratory tract.

## SARS-CoV-2 infection alters the composition of the fecal microbiota in macaques

**Figure 2.**
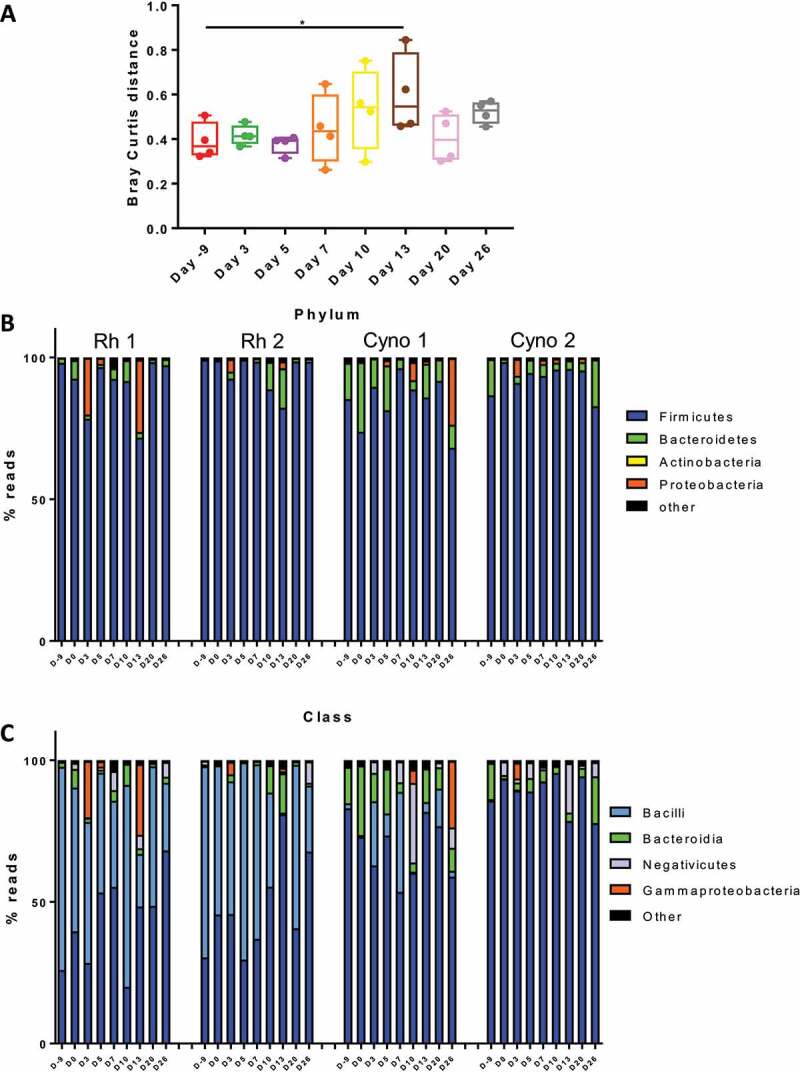
Changes over time in the composition of the bacterial gut microbiota. **A**, Beta diversity was analyzed. Bray Curtis distance between indicated time point and Day 0. *p < .05. The overall composition of the bacterial microbiota at the phylum (b) and class (c) levels was determined for each animal and each time points during the infection (n = 4)

We then turned to analyze the consequences of a SARS-CoV-2 infection on the composition of the gut microbiota. To this end, stool samples were collected prior to and during infection (9 longitudinal samples per animal) and were analyzed using 16S rRNA gene amplicon sequencing. In total, 689,818 sequence reads were analyzed with an average of 19,161 sequence reads per sample (min = 16,433; max = 22,763). Alpha diversity, as assessed by the number of observed species and Shannon indices, did not change significantly over the course of the infection (Supplementary Figure 2a). Beta diversity analysis based on Bray-Curtis index showed clustering per specie and inter-individual variability (Supplementary Figure 2b). However, an analysis of the Bray-Curtis distance on day 0 versus the other time points showed a drift in the fecal microbiota following infection from day 10 and a peak at day 13 (Figure 2a). Of note, β diversity tended to return to basal levels at day 20 and 26 postinfection. A same observation was done when day −9 served as the control (Supplementary Figure 2c). These analyses revealed that SARS-CoV-2 infection induces some alterations in the gut microbiota’s composition.

The fecal microbiota of rhesus macaques and, to a lesser extent, of cynomolgus macaques was dominated by bacteria from the phylum Firmicutes (Figure 2b). Among the other phyla, Bacteroidetes (more prevalent in cynomolgus), and Proteobacteria were detected. With regard to the Firmicutes, we observed some differences at baseline between rhesus macaques and cynomolgus macaques (Figure 2c). Bacteria from the classes Bacilli and Clostridia were the most strongly represented in rhesus macaques, while only Clostridia and Bacteroidia were present in cynomolgus macaques.

**Figure 3.**
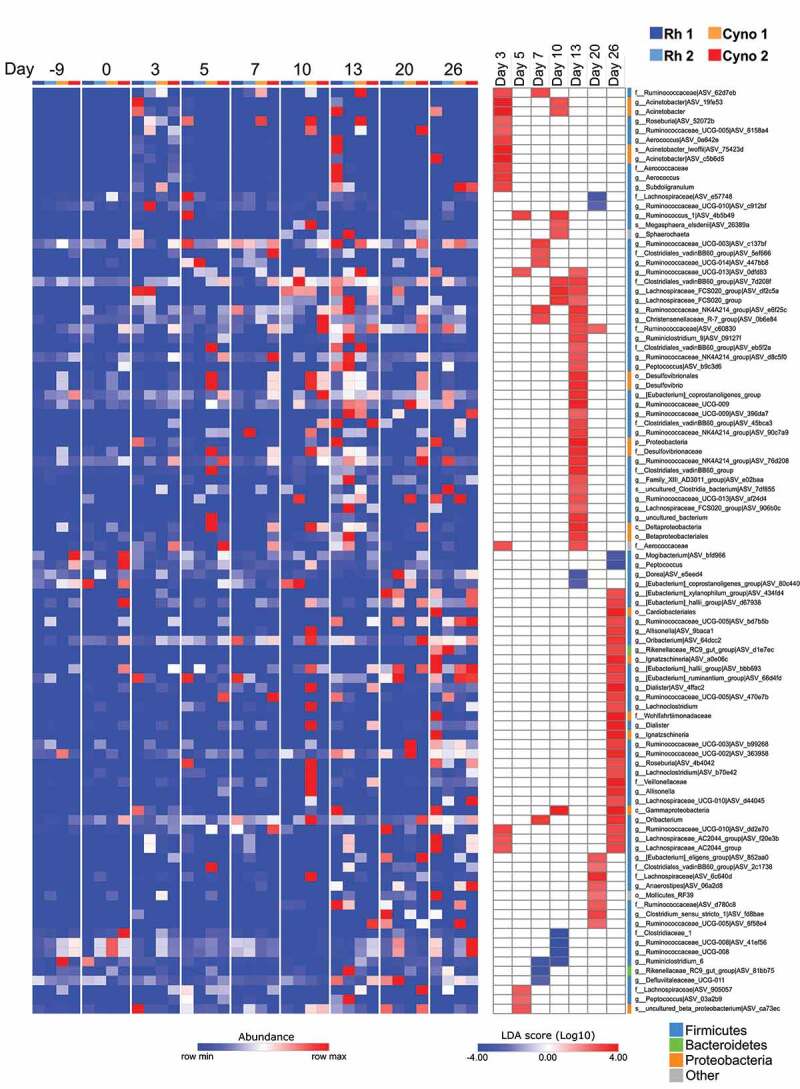
Alterations in the fecal microbiota’s composition over the course of a SARS-CoV-2 infection. A linear discriminant analysis effect size (LEfSe) analysis shows that the representation of the various bacterial taxa changed over the course of the infection. Only taxa with a statistically significant LDA score (log10) > 2 (compared with day −9 & 0) are shown. The heat map on the left shows the relative abundance of the taxa, and the heat map on the right shows the LDA scores. The taxa are clustered by abundance pattern by day compared to D-9 and D0

Phylum- and class-level analyses revealed changes in bacterial composition during infection (Figure 2b). In particular, we observed an increase in the relative abundance of members of the Proteobacteria phylum (mostly from the class Gammaproteobacteria) (Figure 2c) To identify the significant alterations in the gut microbiota at lower taxonomic levels, we compared the composition at each time point post-infection with the two time points before infection (days −9 and 0). Application of the LEfSe pipeline revealed a large number of changes in bacterial taxon abundance during the course of infection – especially 13 dpi (**Figure 3**). Interestingly, some alterations in the fecal microbiota were still present at 26 dpi, although they differed from those observed at earlier time points. Most noticeably, the relative abundance of members of the genus *Acinetobacter* (in the Proteobacteria phylum) increased at early time points. Several other members of the Proteobacteria phylum (notably the Gammaproteobacteria, Betaproteobacteria and Deltaproteobacteria classes) were more abundant at 10 and 13 dpi but also at the later time points. Conversely, the relative abundance of some members of the Firmicutes phylum (notably from the Ruminococcaceae and Lachnospiraceae families) decreased at several time points (**Figure 3**). Although most of the alterations observed at day 10 and 13 were resolved at the later time points, other types of perturbations were observed in post-infection setting at day 26. Overall, SARS-CoV-2 infection in both rhesus macaques and cynomolgus macaques is associated with changes in the composition of the gut microbiota, with a peak at 13 dpi.

## SARS-CoV-2 infection alters the fecal microbiota interaction network

Instead of a simple collection of individual microorganisms, the gut microbiota is rather an ecosystem in which the microorganisms interact together. Networks based on bacterial abundance correlation can be used to explore the complex interplay of microbial communities including potential interdependence or competition between taxa.^41^ The global analysis of the obtained network gives important information on the ecosystem, with a higher number of nodes and connections suggesting a stronger level of interactions within the community.

**Figure 4.**
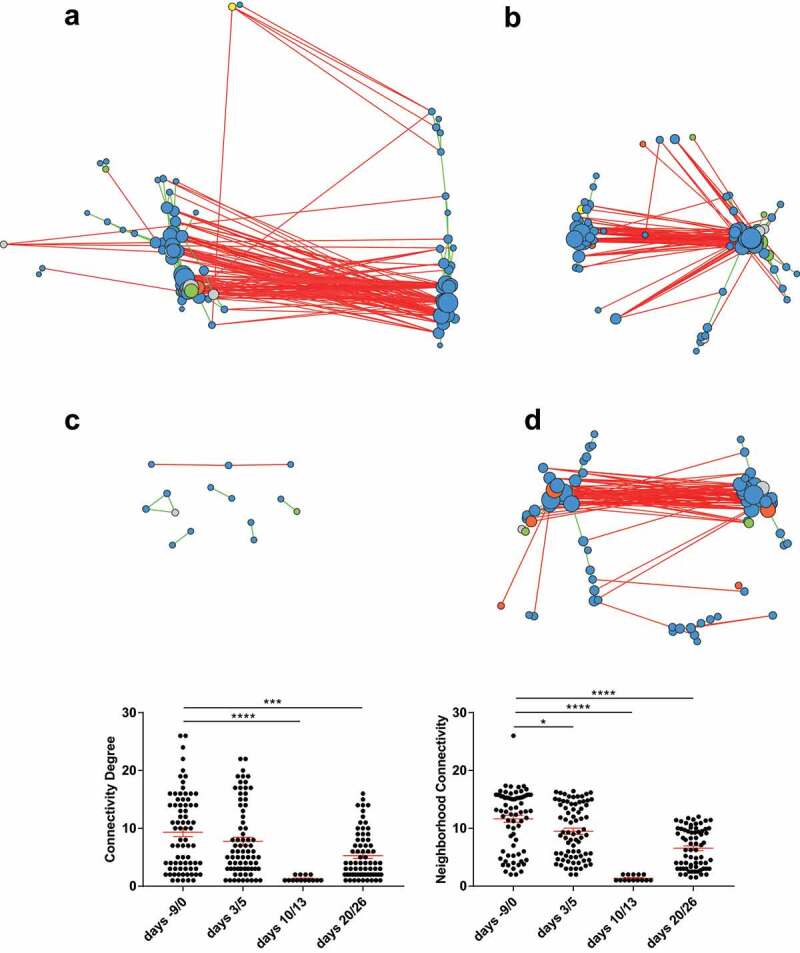
**Alteration of the fecal microbiota interaction network during a SARS-CoV-2 infection**. Genus-level correlation networks for bacterial abundance were built using Spearman’s correlation test for four time periods. **A**, days −9 & 0, before infection. **B**, days 3 & 5 post-infection. **C**, days 10 & 13 post-infection. **D**, day 20 & 26 post-infection. Each circle (node) represents a genus, the color represents the phylum (blue: Firmicutes; green: Bacteroidetes; yellow: Actinobacteria; orange: Proteobacteria; gray: other), and the size increases with the number of direct edges. The edges color indicates the direction of the correlation (green for positive and red for negative). Only significant correlations (*p* < .05 and q < 0.25 after correction for false discovery rate using the Benjamini-Hochberg procedure) are shown. **E**, The mean ± standard error of the mean degree of connectivity and neighborhood connectivity are indicated. *p < .05; ***p < .001; ****p < .0001

We thus built bacterial abundance correlation networks to evaluate the bacterial ecosystem’s structure during the course of SARS-CoV-2 infection. To this end, we grouped together samples from four pairs of successive time points, i.e. days −9 & 0 (before infection), days 3 & 5 (early time points), days 10 & 13 (end of the airway infection), and days 20 & 26 (end of the infection), giving four networks (Figure 4a**-D**). Dense, interconnected networks were obtained for days −9 & 0 and for days 3 & 5. In contrast, the network for days 10 & 13 was highly atrophied, with few nodes and connections. The day 20 & 26 network had a stronger structure with evidence of resilience but was weaker than the initial network. These results were confirmed by a quantitative analysis of the four networks’ degree of connectivity and neighborhood connectivity (Figure 4e). Collectively, these results suggest that COVID-19 infection induces ecological perturbations in the gut microbiota with a peak at 10–13 dpi.

## Fecal microbiota taxa are correlated with features of a SARS-CoV-2 infection

**Figure 5.**
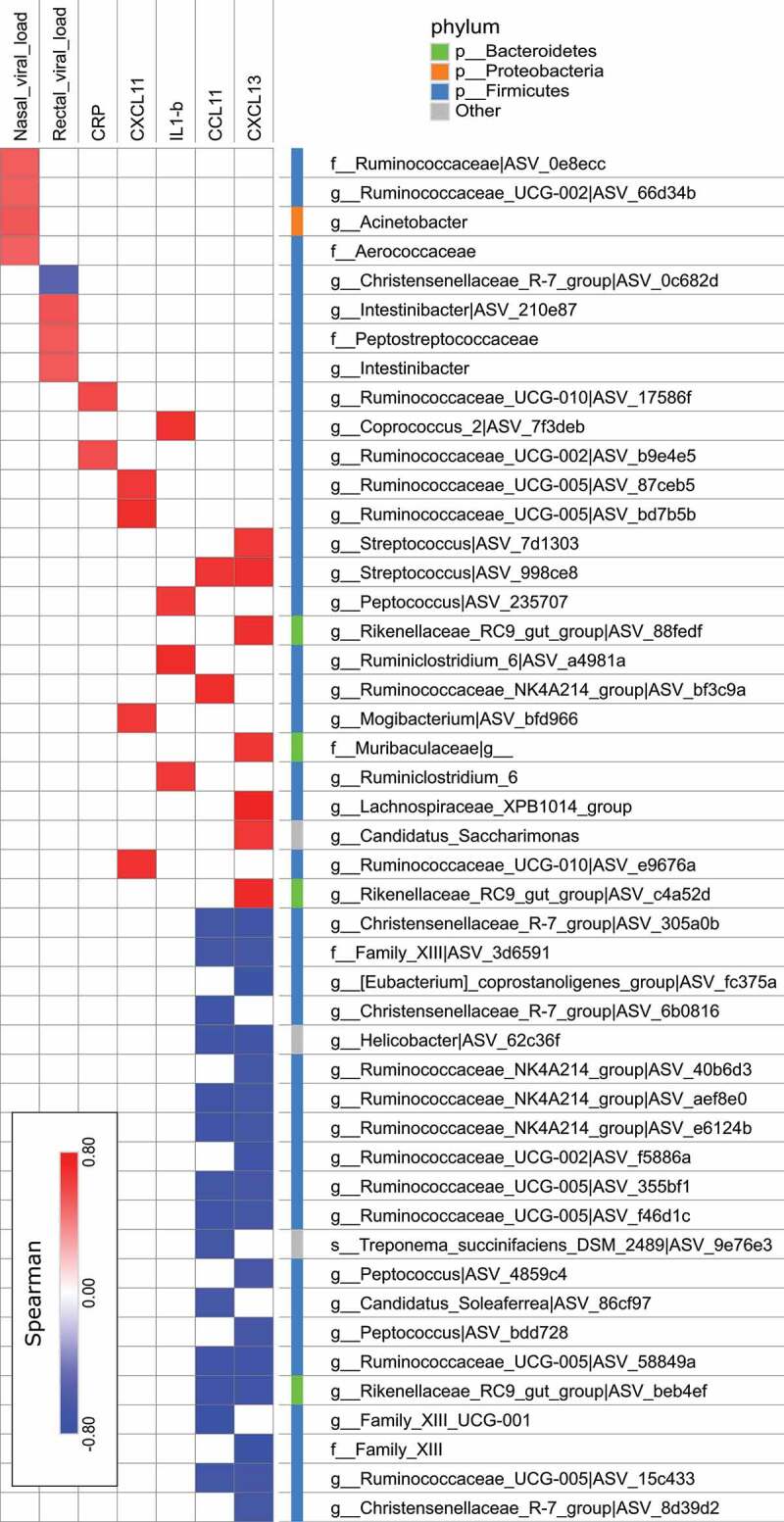
Correlations between bacterial taxa and infection-related variables. Correlation networks were built using Spearman’s correlation and figures were built using Cytoscape V.3.8.0. Only significant correlations (*p* < .05 and q < 0.15 after correction for the false discovery rate, using the Benjamini-Hochberg procedure) are shown

Recent evidence suggests that blood inflammatory factors are correlated with the severity of COVID-19^3,4^ and with changes in the relative abundance of operational taxonomic units.^20,35^ We therefore sought to determine whether changes in gut microbiota’s composition were correlated with infectious parameters, including the viral load and systemic markers (**Figure 5**). Interestingly, the relative abundance of *Acinetobacter* was positively correlated with the presence of SARS-CoV-2 in the nasopharyngeal compartment, while the relative abundance of members of the Peptostreptococcaceae family (notably the *Intestinibacter* genus) was positively correlated with rectal SARS-CoV-2. Only one taxon from the group Christensenellaceae R-7 was negatively correlated with rectal SARS-CoV-2. Moreover, several members of the genus *Streptococcus* were strongly and positively correlated with CCL11 and/or CXCL13 levels, while the opposite correlation was seen with several Firmicutes, notably SCFA producers from the family Ruminococcaceae and the group Christensenellaceae R-7.

## SARS-CoV-2 infection alters the gut microbiota’s metabolic output

To evaluate the functional consequences of the infection-associated changes in the fecal microbiota’s composition, we used a targeted metabolomics approach to quantify three of the most important categories of microbiota-derived metabolites:^29^ SCFAs, bile acids (BA), and tryptophan metabolites.

**Figure 6.**
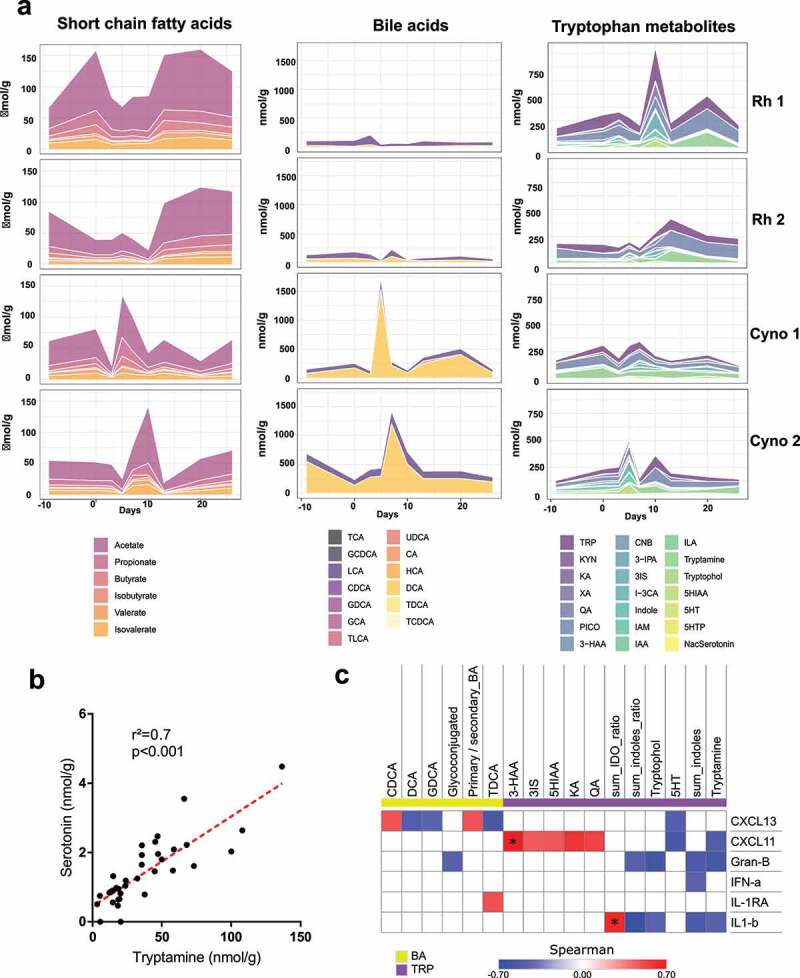
Fecal metabolite production is altered during a SARS-CoV-2 infection. **A**, SCFAs, BAs and tryptophan metabolites were measured in fecal samples from each animal and at each time point, using targeted quantitative metabolomics. Values for individual animals are presented. **B**, Correlations between fecal concentrations of tryptamine and serotonin. **C**, Correlations between fecal metabolites concentrations and plasma cytokine concentrations. Only significant correlations (*p* < .05) are shown. Correlations with q < 0.15 (after correction for the false discovery rate, using the Benjamini and Hochberg procedure) are indicated by an asterisk (*). TCA, taurocholic acid; GCDCA, glycochenodeoxycholic acid; LCA, lithocholic acid; CDCA, chenodeoxycholic acid; GDCA, glycodeoxycholic acid; GCA, glycocholic acid; TLCA, taurocholic acid; UDCA, ursodeoxycholic acid; CA, cholic acid; HCA, hyocholic acid; DCA, deoxycholic acid; TDCA, taurodeoxycholic acid; TCDCA, taurochenodeoxycholic acid; TRP, tryptophan; KYN, kynurenine; KA, kynurenic acid; XA, xanthurenic acid; QA, quinolinic acid; PICO, picolinic acid; 3-HAA, 3-hydroxyanthranilic Acid; CNB, cinnabarinic acid; 3-IPA, 3-Indole propionic acid; 3IS, 3-Indole sulfate; I-3 CA, Indole-3-carboxaldehyde; IAM, indole-3-acetamide; IAA, indole-3-acetic acid; ILA, indole-3-lactic acid; 5HIAA, 5-hydroxyindole acetic acid; 5HT, serotonin; 5HTP, 5-hydroxytryptophan; NasSerotonin, N-Acetylserotonin; sum_indoles, sum of all the metabolites from the indole pathway; sum_IDO, sum of all the metabolites from the IDO pathway; sum_indoles_ratio, sum of all the metabolites from the indole pathway divided by the amount of all the trypophan metabolites; sum_IDO_ratio, sum of all the metabolites from the IDO pathway divided by the amount of all the trypophan metabolites

SCFAs are produced from dietary fibers by some members of the gut microbiota (fermentation) and are key molecules for intestinal barrier and immune and metabolic functions.^29,42–44^ Even though the two macaque species differed slightly with regard to the alterations in fecal metabolites, the overall pattern was the same. As in humans, the dominant SCFAs in macaques are acetate, propionate, and butyrate (see the left panel in Figure 6a for individual values, and Supplementary Figure 3a for the pooled values). Interestingly, the fecal concentrations of these SCFAs changed over the course of infection, particularly between 3 and 13 dpi before going back to the baseline level. In the rhesus macaques, a transient drop of the SCFA concentrations was clearly observed between day 3 and 10 post-infection. In the cynomolgus macaques, the drop of SCFA concentrations was bi-phasic and decreased at 3/5 dpi and 13 dpi. These variations in the fecal SCFA concentration might either reflect changes in their production by bacteria and/or their use by host cells.

Primary BAs are produced by the liver as conjugates and then deconjugated and transformed into secondary BAs by the gut microbiota. BAs are important signaling molecules both within and outside the gastrointestinal tract, notably through their action on nuclear (farnesoid X receptor) and transmembrane (Takeda G-protein receptor 5) receptors.^29,43^ At all time points, the most prominent BAs in stools were the secondary unconjugated BA deoxycholic acid (DCA) and, to a lesser extent in cynomolgus macaques, lithocholic acid (LCA). This suggests that the ability of the microbiota to metabolize BAs was globally conserved during the infection. However, small levels of cholic acid (CA) and chenodeoxycholic acid (CDCA), two primary BAs, were seen mostly at 10 dpi and 13 dpi (Supplementary Figure 4a). Similarly, the global amount of primary BAs and the primary to secondary BAs ratio increased in the stool during the course of the infection and particularly at 10 dpi and 13 dpi (Supplementary Figure 4b), suggesting some alteration in the BA metabolism by the gut microbiota at these timepoints. Interestingly, fecal DCA and LCA concentrations increased in cynomolgus macaques between day 5 and day 13 post-infection and then returned to basal levels (see the middle panel of Figure 6a and Supplementary Figure 3b). At steady state, 95% of the BAs produced in the liver are reabsorbed in the terminal ileum, and so only 5% reaches the colon and can be found in the feces. This increased BA concentration in the feces suggests that reabsorption of the BA in the terminal ileum was impaired – perhaps due to an intrinsic alteration in the ileum’s functions or to accelerated transit. Importantly these altered functions occurred when the strongest microbiota disturbance was observed by 16S rRNA sequencing (day 10–13).

Tryptophan is used by host cells in the gut to produce different types of metabolites via the serotonin and indoleamine 2,3-dioxygenase (IDO) pathways. This amino acid is also metabolized into indoles by the gut bacteria. Indoles are important factors in intestinal homeostasis – notably through their ability to activate the aryl hydrocarbon receptor. Changes in the abundance of several tryptophan metabolites were also observed, mostly between 3 and 13 dpi. An overall increase in the amount of indoles was observed (Figure 6a, right panel, Supplementary Figure 3c, Supplementary Figure 5), although this signal was mainly driven by increased amounts of 3-indole sulfate (3IS) and tryptamine. Interestingly, tryptamine is known to accelerate gastrointestinal transit through a variety of mechanisms. It induces the release of serotonin (5-hydroxytryptamine, 5HT) by enterochromaffin cells in the gut,^45,46^ which in turn stimulates the gastrointestinal motility.^44^ Accordingly, we found that the concentrations of metabolites from the serotonin pathway increased in parallel with that of tryptamine (Supplementary Figure 3), and that there was a strong correlation between the abundances of tryptamine and serotonin (Figure 6b). It was recently shown that tryptamine can activate the serotonin receptor 4 and stimulate fluid secretion in the proximal colon.^47^ We also observed an increased in the abundance of metabolites from the IDO pathway in general and the latter’s end products (quinolinic and picolinic acids) in particular (Figure 6a right panel, Supplementary Figure 3c). This overactivation of the IDO pathway testifies the presence of intestinal inflammation.^44^ Accordingly, total levels of IDO pathway metabolites and the levels of some individual metabolites (such as quinolinic and 3-hydroxyanthranilic acids) were positively correlated with systemic levels of proinflammatory cytokines (Figure 6c). The opposite signal was seen for several indoles and serotonin (5HT). Overall, these data demonstrate that changes in the gut microbiota’s composition during a SARS-CoV-2 infection associates with alteration of bacteria’s metabolic output.

## Discussion

Coronavirus disease 2019 threatens public health and the economy worldwide. The manifestations range from the absence of symptoms to an ARDS syndrome and fatal multi-organ failure. Although the lung is the main viral target, COVID-19 affects many organ systems – including the gastrointestinal tract. It is therefore very important to analyze the impact of a SARS-CoV-2 infection on the gut microbiota and the latter’s critical functions in human health.

Several studies have reported changes in the composition of the gut microbiota in humans with COVID-19.^20,31–35^ Although these clinical studies are informative and have identified operational taxonomic units associated with SARS-CoV-2 infectivity and disease severity, they have various limitations. Firstly, the disease phase at the time of sample collection is not always known. Secondly, samples are not collected at baseline, before infection. Thirdly, in general, the studies lacked longitudinal data and thus did not determine whether or not the microbiotic changes persisted after recovery from COVID-19. Fourthly, the gut microbiota’s composition is influenced by clinical management. Lastly, the microbiota’s products and functional status were not explored in metabolomic studies. The latter type of analysis might be instrumental in understanding microbiota-host interactions and their consequences on the disease outcome. In the present study, we took advantage of nonhuman primate systems – the closest to humans phylogenetically – and evaluated the consequences of a SARS-CoV-2 infection on the composition and metabolic output of the gut microbiota. The use of experimental models to evaluate the effect of an infection on the gut microbiota has several advantages over the analysis of human samples. Firstly, the time points of sample collection (pre-infection, infection, post-infection) can be clearly defined. Secondly, different types of samples can be collected regularly while complying with ethical rules. Changes in the gut microbiota during a viral respiratory infection have been extensively studied in mouse models but not, surprisingly, in nonhuman primates. Furthermore, the impact of an experimental SARS-CoV-2 infection on the gut microbiota had not been previously addressed. In the present study, we decided to assess rhesus macaques and cynomolgus macaques, both currently used as SARS-CoV-2 infection models.^36–40^ Nonhuman primates share various behavioral, physiological and genetic similarities with humans as well as common microbial components. Nonhuman primates and humans also harbor specific microbial signatures.^48,49^ At steady state, and as reported,^50^ we found that rhesus macaques and cynomolgus macaques, which are closely related macaque species, differed with regard to the composition of their microbiota. However, in both macaque species, we observed clear alterations with a peak at 13 dpi, after SARS-CoV-2 had been cleared from the upper airways. This late change was surprising because the peak occurs earlier in the infection (7 to 9 dpi) in mouse models of viral respiratory infection, such as influenza.^18,19,21,22,24–26,51^ It is also noteworthy that although the gut microbiota showed evidence of resilience at later time points (20/26 dpi), some differences persisted after the resolution of the infection, in line with Yeoh and colleagues.^35^ This prolonged alteration might have a role in the post-COVID-19 symptoms that are currently being reported in patients.^52^

The changes in the fecal microbiota’s composition occurred mostly at the family taxonomic level and below. Interestingly, some changes correlated with viral loads and systemic cytokine levels. For instance, the abundances of the family Peptostreptococcaceae and the genus *Intestinibacter* were positively correlated with the presence of SARS-CoV-2 in rectal swabs, whilst one taxa from the group Christensenellaceae R-7 was negatively correlated with rectal SARS-CoV-2. This differs with other reports in which patients with COVID-19 showed (i) a low abundance of SCFA producers, including *Parabacteroides merdae*, Bacteroides (*stercoris, dorei, thetaiataomicron, massiliensis* and *ovatus), Alistipes onderdonkii*, and (ii) the presence of Lachnospiraceae was associated with higher viral loads in fecal samples.^33,34^ In the present study, we noted a positive correlation between several Streptococcus taxa and the amounts of virus-elicited chemokines. This finding is in line with recent human data on a positive association between *Streptococcus infantis* and *Streptococcus parasanguinis* and high SARS-CoV-2 infectivity.^33,34^ In contrast, we observed that the abundance of several taxa known to be SCFA producers (mostly from the Ruminococcaceae family) was negatively correlated with systemic inflammatory markers. The latter data are in line with Zuo et al.’s finding that a low abundance of butyrate producers (*Faecalibacterium prausnitzii, Roseburia*) was negatively correlated with the severity COVID-19.^33^ In Zuo et al.’s study, *Coprobacillus, Clostridium ramosum*, and *Clostridium hathewayi* were also correlated with COVID-19 severity. Lastly, the increase abundance of Proteobacteria over the course of infection was positively correlated with other markers of infection. In line, certain taxa from the phylum Proteobacteria (*Morganella Morganii*) are positively associated with high SARS-CoV-2 infectivity^34^ and with disease severity (*Morganella Morganii* and *Enterobacter cloacae*) in patients with COVID-19.^33^

In the present study, changes in gut microbiota’s composition during experimental COVID-19 translated into several metabolic changes. The SCFA levels were altered during SARS-CoV-2 infection, particularly between day 3 and 13. Changes in SCFA production have been observed during experimental influenza.^22^ However, it is not yet known whether this alteration impacts gut homeostasis (e.g., inflammation and barrier properties) and secondary systemic outcomes; this topic warrants future studies. Our recent data show that the drop of SCFAs during influenza infection lowers pulmonary defenses and favors secondary bacterial infection in mice.^22^ The present study is the first to have shown that a respiratory viral infection impacts BA and tryptophan metabolisms. A slight impairment in BA transformation was evidenced at 13 dpi, i.e. concomitantly with the greatest changes in bacterial composition. Moreover, the primary-to-secondary BA ratio (a marker of BA transformation by the gut microbiota) was positively correlated with serum levels of chemokines such as CXCL13. We also observed an increase in the fecal BA level, suggesting that the infection leads to accelerated transit and/or impaired BA reabsorption in the ileum. An increase in the fecal concentration of the tryptophan metabolite tryptamine (known to accelerate transit^44–46^) was also observed and might have been involved in the increase in fecal BA output. The concomitant overall increase in metabolites from the IDO pathway suggests that the intestine was inflamed to some extent during the course of the infection. Accordingly, the levels of certain individual IDO metabolites and the cumulative levels of all IDO metabolites were positively correlated with systemic markers of inflammation. Conversely, some metabolites from the two other tryptophan metabolism pathways (including 5HT, tryptophol, and tryptamine) were negatively correlated with the markers of inflammation. It remains to be seen whether these alterations in BA and tryptophan metabolism are involved in the severity of a SARS-CoV-2 infection.

The present study is the first to have evidenced alterations in the gut microbiota’s composition and functional activity during an experimental SARS-CoV-2 infection. Relative to influenza infection in mice,^18–26^ alteration of the gut microbiota communities was less marked and most changes were observed at low taxonomic levels. This might be due to the low level of pathogenicity of SARS-CoV-2 in the nonhuman primate system. Indeed, macaques infected with SARS-CoV-2 develop only a mild illness, showing signs of pneumonia similar to those in humans with COVID-19 but without adverse clinical symptoms.^36–40^ It should be noted that the current study was a pilot study with limitations including the low number of animals used. Regarding the heterogeneity across animals, both in terms of clinical outcomes (viral load, blood markers) and gut microbiota composition at baseline (as seen in humans), the data provided in the current study need confirmation with a higher number of animals. Another limitation lies on the correlation analysis. Indeed, it comprised a lot of variables (relative abundance of many taxa, at many levels) and not independent markers of COVID infection were analyzed. Although our findings provide guidance into future research, the real connection between bacterial taxa and disease markers need to be established. In particular, it will now be important to determine whether these alterations influence the gastrointestinal and pulmonary signs and symptoms of COVID-19. The development of relevant models of both low pathogenicity and high pathogenicity (i.e., mice and hamsters) will be instrumental in these investigations.^53^ If the severity and lethality of COVID-19s are modulated by the gut microbiota, targeting the latter’s various components might constitute an attractive therapeutic strategy.

## Materials and Methods

### Ethics and biosafety statement

Young adult, 3–5 years of age, female cynomolgus macaques (*Macaca fascicularis*) (3 years-old) and rhesus macaques (*Macaca mulatta*) (5 years-old), and originating from Mauritian and Chinese AAALAC certified breeding centers, respectively, were used in this study. All animals were housed in Infectious Diseases Models for Innovative Therapies (IDMIT) infrastructure facilities (CEA, Fontenay-aux-roses), under BSL-3 containment (Animal facility authorization #D92-032-02, Prefecture des Hauts de Seine, France) and in compliance with European Directive 2010/63/EU, the French regulations and the Standards for Human Care and Use of Laboratory Animals, of the Office for Laboratory Animal Welfare (OLAW, assurance number #A5826-01, US). All animal procedures were in accordance with the institutional animal care and use committee (CEEA-044, Paris, France) under statement number A20-011. The study was authorized by the “Research, Innovation and Education Ministry” under registration number APAFIS#24434-2020030216532863v1.

## Reagents

Methanol (MeOH), acetonitrile, and formic acid were of HPLC grade. Analytical grade of NaOH, propan-1-ol, pyridine, hexane, and propylchloroformate (PCF) were purchased as well from Sigma-Aldrich (Saint Quentin Fallavier, France). Deionized water comes from a Milli-Q Elix system fitted with a LC-PaK and a MilliPak filter at 0.22 μm (Merck Millipore, Guyancourt, France). The following bile acid standards: cholic acid (CA), chenodeoxycholic acid (CDCA), deoxycholic acid (DCA), lithocholic acid (LCA), ursodeoxycholic acid (UDCA), hyodeoxycholic acid (HDCA), lycocholic acid (GCA), glycochenodeoxycholic acid (GCDCA), glycodeoxycholic acid (GDCA), glycolithocholic acid (GLCA), taurocholic acid (TCA), taurochenodeoxycholic acid (TCDCA), taurodeoxycholic acid (TDCA), tauroursodeoxycholic acid (TUDCA), and taurolithocholic acid (TLCA) and ammonium acetate were purchased from Sigma Chemical (St. Louis, MO, USA). All tryptophan reference, isotope-labeled metabolites and SCFAs (acetate, propionate, butyrate, isobutyrate, valerate, isovalerate, acetate-D3, butyrate-13C2 and valerate-D9) were purchased from Sigma-Aldrich (Saint Quentin Fallavier, France). The stock solutions of bile acids, tryptophan metabolite and SCFAs were prepared separately in methanol at the concentration of 10 mmol/l, and the stock solutions were stored at −20°C.

## Virus, animals and sample collection

The virus strain used in this study (hCoV-19/France/lDF0372/2020 strain) was isolated by the National Reference Center for Respiratory Viruses (NRC-VIR, Institut Pasteur, Paris, France) from a nasopharyngeal swab from one of the first French cases.^54, **55^ Production of viral stock in Vero E6 cells is described in.^38^ Animals were exposed to a total dose of 2 × 10^7^ PFUs of SARS-CoV-2 *via* the combination of intranasal and intra-tracheal routes. This procedure allowed infection in the upper and lower respiratory tracts. Atropine (0.04 mg/kg) was used for pre-medication and ketamine (5 mg/kg) with medetomidine (0.042 mg/kg) were used for anesthesia. To follow the kinetic of viral replication, nasopharyngeal, tracheal and rectal fluids (swabs) were collected at base line, 3, 5, 7, 10, 13, 20, and 26 dpi. To study the SARS-CoV-2 infection’s impact on the gut microbiota, feces from each challenged macaque were sampled 9 days before infection, the day of infection (day 0, 1 h before) to characterize baseline microbiota composition, and at 3, 5, 7, 10, 13, 20, and 26 dpi. Samples collected at day −9 and at day 0 served as references to identify potential changes in microbiota composition following infection. Fecal samples were stored at −80°C until further analysis. Blood samples were collected at baseline and at several time points during the course of infection with the limit of 7.5% of blood body volume per week.

## Virus quantification in nonhuman primates

Upper respiratory (nasopharyngeal and tracheal) and rectal specimens were collected with swabs as described.^38^ Relative quantification of the viral genome was performed by one-step real-time quantitative reverse transcriptase and polymerase chain reaction (RT–qPCR) from viral RNA extracted using the Extraction NucleoSpin Dx Virus kit (Macherey Nagel). Primers and probes (RdRp_nCoV_IP4) were designed by the NRC-VIR to target the RdRp gene spanning nt 14010–14116. Real-time one-step RT-qPCR was performed with the Superscript III one-step RT-PCR system with Platinum Taq Polymerase (Invitrogen, Darmstadt, Germany). Thermal cycling was performed at 55°C for 20 min for reverse transcription, followed by 95°C for 3 min and then 50 cycles of 95°C for 15 s, 58°C for 30 s, on a Light Cycler 480 (96) thermocycler (Roche)^36^].

## Plasma cytokine quantification

Cytokines were quantified in EDTA-treated plasma using nonhuman primate ProcartaPlex immunoassay (ThermoFisher Scientific) and a Bioplex 200 analyzer (Bio-Rad) according to manufacturer’s instructions.

## Genomic DNA extraction and sequencing

Fecal Genomic DNA was extracted from 200 mg of feces as previously described.^56^ Following microbial lysis with both mechanical and chemical steps, nucleic acids were precipitated in isopropanol for 10 minutes at room temperature, incubated for 15 minutes on ice and centrifuged for 30 minutes at 15,000 *g* and 4°C. Pellets were suspended in 112 µl of phosphate buffer and 12 µl of potassium acetate. After RNase treatment and DNA precipitation, nucleic acids were recovered via centrifugation at 15,000 *g* and 4°C for 30 min. The DNA pellet was suspended in 100 µl of TE buffer. Microbial diversity and composition were determined for each sample by targeting a portion of the ribosomal genes. A 16S rRNA gene fragment comprising V3 and V4 hypervariable regions (16S; 5′-TACGGRAGGCAGCAG-3′ and 5′-CTACCNGGGTATCTAAT-3′) was amplified using an optimized and standardized 16S-amplicon-library preparation protocol (Metabiote, GenoScreen). Briefly, 16S rRNA gene PCR was performed starting with 5 ng genomic DNA and using unique barcoded primers (Metabiote MiSeq Primers, GenoScreen) at final concentrations of 0.2 μM and an annealing temperature of 50°C f or 30 cycles. The PCR products were purified using an Agencourt AMPure XP-PCR Purification system (Beckman Coulter), quantified according to the manufacturer’s protocol, and multiplexed at equal concentrations. Sequencing was performed using a 250-bp paired-end sequencing protocol on an Illumina MiSeq platform (Illumina) at GenoScreen. Positive (artificial bacteria Community comprising 17 different bacteria (ABCv2)) and negative (sterile water) control were also included.

## Gut Microbiota analysis

Following DNA extraction and sequencing, raw paired-end reads were processed in a data curation pipeline, that includes a step of removal of low quality reads (Qiime2 2019.10).^57^ Remaining sequences were assigned to samples based on barcode matches, and barcode and primer sequences were then trimmed. The sequences were denoized using the DADA2 method, and reads were classified using Silva reference database (version 132).^58^ Alpha and beta diversity were computed using the phyloseq package (v1.24.2).^59^ Principal Coordinate analyses of the Bray Curtis distance and Jaccard index were performed to assess beta diversity. The number of observed species, Chao1, Shannon and Simpson indexes were calculated using rarefied data (depth = 24,000 sequences/sample) and used to characterize alpha diversity. Raw sequence data are accessible in the European Nucleotide Archive (accession number:). Differential analysis was performed using the linear discriminant analysis effect size (LEfSe) pipeline.^60^ Correlation networks were built using Spearman’s correlation and figures were built using Cytoscape V.3.8.0. The connectivity degree of a node is defined by the number of edges of the node. The neighborhood connectivity of a node is defined as the average connectivity of all neighbors of the node.

## Metabolomic analysis of fecal samples

Fecal samples (1 g) from nonhuman primates were inactivated by 1.6 ml MeOH. After homogenization, 3 aliquots (450 µL in each vial) were taken for bile acid, tryptophan metabolites and SCFA analysis, respectively. Extraction steps were carried out at 4°C to avoid the degradation of compounds, especially for SCFAs. Samples were extracted and analyzed as follows. *Tryptophan metabolites*: 50 µl internal standard in MeOH and 500 µl H_2_O were added to the 450 µl fecal extracts. After 20 min, agitation at 4°C the samples were centrifugated at 12,000 *g* and 450 µl MeOH were added to the pellet for homogenization and centrifugation. The two supernatants were pooled and evaporated under nitrogen. The dried residues were dissolved in 600 µl MeOH and 3 µl were injected. The LC-MS/MS procedure was performed as previously described.^61^ Mass spectra were obtained using an 5500 Q-Trap (Sciex, Concord, Ontario, Canada) equipped with a TurboIon electrospray (ESI) interface set in the negative mode (needle voltage +5000 V) with nitrogen as the nebulizer set at 40 (arbitrary pressure unit given by the equipment provider). Curtain and heater pressures were set at 20 and 40, respectively (arbitrary unit). The ion source temperature was set at 350°C. Declustering and entrance potentials were set at +60 V and +10 V, respectively. The MS/MS detection was operated at unit/unit resolution. The acquisition dwell time for each transition monitored was 25 ms. Data were acquired by the Analyst® software (version 1.6.2, -Sciex) in the Multiple Reaction Monitoring (MRM) mode. *Bile acid molecular species*. 2 ml of 0.2 M NaOH were added to the 450 µl fecal methanol extracts. After 20 min agitation at 60°C, 4 ml H_2_O containing internal standard were added. The whole samples were centrifuged at 12,000 *g* and the supernatants were collected. Final extraction and Mass spectrometry analysis were performed as already described.^62^ *SCFAs*: sample preparation was adapted from protocol of Zheng and collaborators.^63^ Approximatively 30 mg of fecal samples were used and suspended with 1050 µl of a solution of NaOH at 0.005 M including internal standard mix of acetate-D3, butyrate-13 C2 and valerate-D9 at 61 µM and ceramic beads. Samples were homogenized at 6500 rpm, 3 × 20 s using Prescellys® Evolution (Bertin Technologies, Montigny-le-Bretonneux, France). 300 µl of each supernatant were collected and transferred to 5 ml glass tube. 500 µl of propanol/pyridine mix (3:2 v/v) were added and then vortexed. 50 µl of PCF was successively added twice to the solution and vortexed. Mixtures were sonicated and centrifuged at 2000 x g and 4°C during 5 min. 200 µl of organic phase were transferred to GC/MS vials before their injections. SCFAs in fecal samples were quantified by Gas Chromatographic/mass spectrometry using an ISQ LT™ equipped with a Triplus RSH (Thermo Fisher Scientific, Illkirch, France). A fused-silica capillary column with a (5%-phenyl)-methylpolysiloxane phase (DB-5 ms, J&W Scientific, Agilent Technologies Inc., USA) of 50 m x 0.25 mm i.d coated with 0.25 µm film thickness was used. Temperatures of the front inlet, MS transfer line, and electron impact ion source were set at 260, 290, and 230°C, respectively. Helium was supplied with carrier gas at a flow rate of 1 ml/min. Oven temperature was set initially at 50°C during 1.5 min. Temperature was raised to 70at 8°C/min and to 85°C at 6°C/min. Then, temperature was successively elevated to 110°C at 22°C/min and to 120 at 12°C/min. Oven temperature was finally set to 300at 125°C/min and held 3 min. The run time was 15 min in targeted SIM mode. Injected sample volume was set to 1 µl in split mode with a 20:1 ratio. Data processing was performed using Xcalibur® software (version 3.0, Thermofisher Scientific, Illkirch, France)

## Statistical analysis

Statistical analysis was performed in the R statistical environment (R version 3.6.2). When appropriate, statistical analyses were performed using the two-tailed non-nonparametric Mann-Whitney test or Kruskal–Wallis test with Dunn’s multiple comparison test. In all statistical analyses, differences with *p* values <.05 were considered significant. The *p* values were corrected using the Benjamini and Hochberg procedure to control for the false discovery rate. Microbiota-specific analysis is described in the Gut Microbiota analysis section.

## Supplementary Material

Supplemental Material

## Data Availability

Please contact author for data requests

## Abbreviations

COVID-19, Coronavirus disease 2019; SARS-CoV-2, severe acute respiratory syndrome coronavirus 2; acute respiratory distress syndrome (ARDS); SCFAs, short-chain fatty acids; PFUs, plaque forming units; dpi, day postinfection; CRP, C reactive protein; IL, interleukin, CCL, C-C motif chemokine ligand; CXCL, chemokine (C-X-C motif) ligand; IDO, indoleamine 2,3-dioxygenase; BA, bile acid; DCA, deoxycholic acid; LCA, lithocholic acid; CA, cholic acid; CDCA, 3IS, 3-indole sulfate. 5HT, 5-hydroxytryptamine.

## References

[1] Zhu N, Zhang D, Wang W, Li X, Yang B, Song J, Zhao X, Huang B, Shi W, Lu R, et al. A Novel Coronavirus from patients with pneumonia in China, 2019. N Engl J Med. 2020;382(8):727–19. doi:10.1056/NEJMoa2001017. 31978945 PMC7092803

[2] Chen T, Wu D, Chen H, Yan W, Yang D, Chen G, Ma K, Xu D, Yu H, Wang H, et al. Clinical characteristics of 113 deceased patients with coronavirus disease 2019: retrospective study. BMJ. 2020;368:m1091. 32217556 10.1136/bmj.m1091 PMC7190011

[3] Huang C, Wang Y, Li X, Ren L, Zhao J, Hu Y, Zhang L, Fan G, Xu J, Gu X, et al. Clinical features of patients infected with 2019 novel coronavirus in Wuhan, China. Lancet. 2020;395:497–506. 31986264 10.1016/S0140-6736(20)30183-5 PMC7159299

[4] Ruan Q, Yang K, Wang W, Jiang L, Song J. Clinical predictors of mortality due to COVID-19 based on an analysis of data of 150 patients from Wuhan, China. Intensive Care Med. 2020;46:846–848. 32125452 10.1007/s00134-020-05991-x PMC7080116

[5] Esteve A, Permanyer I, Boertien D, Vaupel JW. National age and coresidence patterns shape COVID-19 vulnerability. Proc Natl Acad Sci USA. 2020;117:16118–16120. 32576696 10.1073/pnas.2008764117 PMC7368248

[6] Guan W-J, Ni Z-Y, Hu Y, Liang W-H, Ou C-Q, He J-X, Liu L, Shan H, Lei C-L, Hui DSC, et al. Clinical Characteristics of Coronavirus Disease 2019 in China. N Engl J Med. 2020;382:1708–1720. 32109013 10.1056/NEJMoa2002032 PMC7092819

[7] Jordan RE, Adab P, Cheng KK. Covid-19: risk factors for severe disease and death. BMJ. 2020;368:m1198. 32217618 10.1136/bmj.m1198

[8] Yang J, Zheng Y, Gou X, Pu K, Chen Z, Guo Q, Ji R, Wang H, Wang Y, Zhou Y. Prevalence of comorbidities and its effects in patients infected with SARS-CoV-2: a systematic review and meta-analysis. Int J Infect Dis. 2020;94:91–95. 32173574 10.1016/j.ijid.2020.03.017 PMC7194638

[9] Zhou F, Yu T, Du R, Fan G, Liu Y, Liu Z, Xiang J, Wang Y, Song B, Gu X, et al. Clinical course and risk factors for mortality of adult inpatients with COVID-19 in Wuhan, China: a retrospective cohort study. Lancet. 2020;395:1054–1062. 32171076 10.1016/S0140-6736(20)30566-3 PMC7270627

[10] Bastard P, Rosen LB, Zhang Q, Michailidis E, Hoffmann -H-H, Zhang Y, Dorgham K, Philippot Q, Rosain J, Béziat V, et al. Auto-antibodies against type I IFNs in patients with life-threatening COVID-19. Science. 2020;370(6515):eabd4585. 32972996 10.1126/science.abd4585 PMC7857397

[11] Zhang Q, Bastard P, Liu Z, Le Pen J, Moncada-Velez M, Chen J, Ogishi M, Sabli IKD, Hodeib S, Korol C, et al. Inborn errors of type I IFN immunity in patients with life-threatening COVID-19. Science. 2020;370(6515):eabd4570. 32972995 10.1126/science.abd4570 PMC7857407

[12] Trottein F, Sokol H. Potential causes and consequences of gastrointestinal disorders during a SARS-CoV-2 infection. Cell Rep. 2020;32:107915. 32649864 10.1016/j.celrep.2020.107915 PMC7332457

[13] Mao R, Qiu Y, He J-S, Tan J-Y, Li X-H, Liang J, Shen J, Zhu L-R, Chen Y, Iacucci M, et al. Manifestations and prognosis of gastrointestinal and liver involvement in patients with COVID-19: a systematic review and meta-analysis. Lancet Gastroenterol Hepatol. 2020;5:667–678. 32405603 10.1016/S2468-1253(20)30126-6 PMC7217643

[14] Lamers MM, Beumer J, Van Der Vaart J, Knoops K, Puschhof J, Breugem TI, Ravelli RBG, Paul Van Schayck J, Az M, Hq D, et al. SARS-CoV-2 productively infects human gut enterocytes. Science. 2020;369:50–54. 32358202 10.1126/science.abc1669 PMC7199907

[15] Zhou J, Li C, Liu X, Chiu MC, Zhao X, Wang D, Wei Y, Lee A, Zhang AJ, Chu H, et al. Infection of bat and human intestinal organoids by SARS-CoV-2. Nat Med. 2020;26:1077–1083. 32405028 10.1038/s41591-020-0912-6

[16] Xiao F, Tang M, Zheng X, Liu Y, Li X, Shan H. Evidence for gastrointestinal infection of SARS-CoV-2. Gastroenterology. 2020;158:1831–1833. 32142773 10.1053/j.gastro.2020.02.055 PMC7130181

[17] Blander JM, Longman RS, Iliev ID, Sonnenberg GF, Artis D. Regulation of inflammation by microbiota interactions with the host. Nat Immunol. 2017;18:851–860. 28722709 10.1038/ni.3780 PMC5800875

[18] Bartley JM, Zhou X, Kuchel GA, Weinstock GM, Haynes L. Impact of Age, Caloric restriction, and influenza infection on mouse gut microbiome: an exploratory study of the role of age-related microbiome changes on influenza responses. Front Immunol. 2017;8:1164–1174. 28979265 10.3389/fimmu.2017.01164 PMC5611400

[19] Deriu E, Boxx GM, He X, Pan C, Benavidez SD, Cen L, Rozengurt N, Shi W, Cheng G. Influenza virus affects intestinal microbiota and secondary *Salmonella* infection in the gut through type I interferons. PLoS Pathog. 2016;12:e1005572. 27149619 10.1371/journal.ppat.1005572 PMC4858270

[20] Gu S, Chen Y, Wu Z, Chen Y, Gao H, Lv L, Guo F, Zhang X, Luo R, Huang C, et al. Alterations of the gut microbiota in patients with COVID-19 or H1N1 influenza. Clin Infect Dis. 2020;71:2669–2678. 32497191 10.1093/cid/ciaa709 PMC7314193

[21] Qin N, Zheng B, Yao J, Guo L, Zuo J, Wu L, Zhou J, Liu L, Guo J, Ni S, et al. Influence of H7N9 virus infection and associated treatment on human gut microbiota. Sci Rep. 2015;5:14771–14782. 26490635 10.1038/srep14771 PMC4614822

[22] Sencio V, Barthelemy A, Tavares LP, Machado MG, Soulard D, Cuinat C, Queiroz-Junior CM, Noordine M-L, Salomé-Desnoulez S, Deryuter L, et al. Gut dysbiosis during influenza contributes to pulmonary pneumococcal superinfection through altered short-chain fatty acid production. Cell Rep. 2020;30:2934–2947. 32130898 10.1016/j.celrep.2020.02.013

[23] Singhal R, Shah YM. Oxygen battle in the gut: hypoxia and hypoxia-inducible factors in metabolic and inflammatory responses in the intestine. J Biol Chem. 2020;295:10493–10505. 32503843 10.1074/jbc.REV120.011188 PMC7383395

[24] Wang J, Li F, Wei H, Lian Z-X, Sun R, Tian Z. Respiratory influenza virus infection induces intestinal immune injury via microbiota-mediated Th17 cell-dependent inflammation. J Exp Med. 2014;211:2397–2410. 25366965 10.1084/jem.20140625 PMC4235643

[25] Yildiz S, Mazel-Sanchez B, Kandasamy M, Manicassamy B, Schmolke M. Influenza A virus infection impacts systemic microbiota dynamics and causes quantitative enteric dysbiosis. Microbiome. 2018;6:9. 29321057 10.1186/s40168-017-0386-z PMC5763955

[26] Zhang Q, Hu J, Feng J-W, Hu X-T, Wang T, Gong W-X, Huang K, Guo Y-X, Zou Z, Lin X, et al. Influenza infection elicits an expansion of gut population of endogenous *Bifidobacterium animalis* which protects mice against infection. Gen Biol. 2020;21:99–125. 10.1186/s13059-020-02007-1 PMC7187530 32345342

[27] Dabke K, Hendrick G, Devkota S. The gut microbiome and metabolic syndrome. J Clin Invest. 2019;129:4050–4057. 31573550 10.1172/JCI129194 PMC6763239

[28] Helmink BA, Khan MAW, Hermann A, Gopalakrishnan V, Wargo JA. The microbiome, cancer, and cancer therapy. Nat Med. 2019;25:377–388. 30842679 10.1038/s41591-019-0377-7

[29] Lavelle A, Sokol H. Gut microbiota-derived metabolites as key actors in inflammatory bowel disease. Nat Rev Gastroenterol Hepatol. 2020;17:223–237. 32076145 10.1038/s41575-019-0258-z

[30] Kruglikov IL, Shah M, Scherer PE. Obesity and diabetes as comorbidities for COVID-19: underlying mechanisms and the role of viral-bacterial interactions. eLife. 2020;9:e61330. 32930095 10.7554/eLife.61330 PMC7492082

[31] Gou W, Fu Y, Yue L, Chen G, Cai X, Shuai M, Xu F, Yi X, Chen H, Zhu YJ, et al. Gut microbiota may underlie the predisposition of healthy individuals to COVID-19. *medRxiv*. http://medrxiv.org/lookup/doi/10.1101/2020.04.22.20076091.

[32] Yu L, Tong Y, Shen G, Fu A, Lai Y, Zhou X, Yuan Y, Wang Y, Pan Y, Yu Z, et al. Immunodepletion with hypoxemia: a potential high risk subtype of coronavirus disease 2019. 2020. *medRxiv*. 10.1101/2020.03.03.20030650.

[33] Zuo T, Zhan H, Zhang F, Liu Q, Tso EY, Lui GC, Chen N, Li A, Lu W, Chan FK, et al. Alterations in fecal fungal microbiome of patients with COVID-19 during time of hospitalization until discharge. Gastroenterology. 2020;159:1302–1310. 32598884 10.1053/j.gastro.2020.06.048 PMC7318920

[34] Zuo T, Liu Q, Zhang F, Lui GC, Tso EY, Yeoh YK, Chen Z, Boon SS, Chan FK, Chan PK, et al. Depicting SARS-CoV-2 faecal viral activity in association with gut microbiota composition in patients with COVID-19. Gut. 2020;70:276–284. 32690600 10.1136/gutjnl-2020-322294 PMC7385744

[35] Yeoh Y, Zuo T, Lui G, Zhang F, Liu Q, Li A, Chung A, Cheung C, Tso E, Fung K, et al. Gut microbiota composition reflects disease severity and dysfunctional immune responses in patients with COVID-19. Gut 2021; gutjnl-2020-323020. 10.1136/gutjnl-2020-323020 PMC7804842 33431578

[36] Chandrashekar A, Liu J, Martinot AJ, McMahan K, Mercado NB, Peter L, Tostanoski LH, Yu J, Maliga Z, Nekorchuk M, et al. SARS-CoV-2 infection protects against rechallenge in rhesus macaques. Science. 2020;369:812–817. 32434946 10.1126/science.abc4776 PMC7243369

[37] Deng W, Bao L, Liu J, Xiao C, Liu J, Xue J, Lv Q, Qi F, Gao H, Yu P, et al. Primary exposure to SARS-CoV-2 protects against reinfection in rhesus macaques. Science. 2020;369:818–823. 32616673 10.1126/science.abc5343 PMC7402625

[38] Maisonnasse P, Guedj J, Contreras V, Behillil S, Solas C, Marlin R, Naninck T, Pizzorno A, Lemaitre J, Gonçalves A, et al. Hydroxychloroquine use against SARS-CoV-2 infection in non-human primates. Nature. 2020;585:584–587. 32698191 10.1038/s41586-020-2558-4

[39] Munster VJ, Feldmann F, Williamson BN, Van Doremalen N, Pérez-Pérez L, Schulz J, Meade-White K, Okumura A, Callison J, Brumbaugh B, et al. Respiratory disease in rhesus macaques inoculated with SARS-CoV-2. Nature. 2020;585:268–272. 32396922 10.1038/s41586-020-2324-7 PMC7486227

[40] Rockx B, Kuiken T, Herfst S, Bestebroer T, Lamers MM, Oude Munnink BB, De Meulder D, Van Amerongen G, Van Den Brand J, Okba NMA, et al. Comparative pathogenesis of COVID-19, MERS, and SARS in a nonhuman primate model. Science. 2020;368:1012–1015. 32303590 10.1126/science.abb7314 PMC7164679

[41] Faust K, Sathirapongsasuti JF, Izard J, Segata N, Gevers D, Raes J, Huttenhower C. Microbial co-occurrence relationships in the human microbiome. PLoS Comput Biol. 2012;8:e1002606. 22807668 10.1371/journal.pcbi.1002606 PMC3395616

[42] Tan JK, McKenzie C, Mariño E, Macia L, Mackay CR. Metabolite-sensing G protein-coupled receptors-facilitators of diet-related immune regulation. Annu Rev Immunol. 2017;35:371–402. 28446062 10.1146/annurev-immunol-051116-052235

[43] Molinaro A, Wahlström A, Marschall H-U. Role of bile acids in metabolic control. Trends Endocrinol Metab. 2018;29:31–41. 29195686 10.1016/j.tem.2017.11.002

[44] Agus A, Planchais J, Sokol H. Gut microbiota regulation of tryptophan metabolism in health and disease. Cell Host Microbe. 2018;23:716–724. 29902437 10.1016/j.chom.2018.05.003

[45] Roager HM, Licht TR. Microbial tryptophan catabolites in health and disease. Nat Commun. 2018;9:3294–3393. 30120222 10.1038/s41467-018-05470-4 PMC6098093

[46] Takaki M, Mawe GM, Barasch JM, Gershon MD, Gershon MD. Physiological responses of guinea-pig myenteric neurons secondary to the release of endogenous serotonin by tryptamine. Neuroscience. 1985;16:223–240. 2940472 10.1016/0306-4522(85)90059-4

[47] Bhattarai Y, Williams BB, Battaglioli EJ, Whitaker WR, Till L, Grover M, Linden DR, Akiba Y, Kandimalla KK, Zachos NC, et al. Gut microbiota-produced tryptamine activates an epithelial G-protein-coupled receptor to increase colonic secretion. Cell Host Microbe. 2018;23(775–785.e5). 10.1016/j.chom.2018.05.004 PMC6055526 29902441

[48] McKenna P, Hoffmann C, Minkah N, Aye PP, Lackner A, Liu Z, Lozupone CA, Hamady M, Knight R, Bushman FD. The macaque gut microbiome in health, lentiviral infection, and chronic enterocolitis. PLoS Pathog. 2008;4:e20. 18248093 10.1371/journal.ppat.0040020 PMC2222957

[49] Chen Z, Yeoh YK, Hui M, Wong PY, Chan MCW, Ip M, Yu J, Burk RD, Chan FKL, Chan PKS. Diversity of macaque microbiota compared to the human counterparts. Sci Rep. 2018;8:15573. 30349024 10.1038/s41598-018-33950-6 PMC6197227

[50] Cui Y-F, Wang F-J, Yu L, Ye -H-H, Yang G-B. Metagenomic comparison of the rectal microbiota between rhesus macaques (Macaca mulatta) and cynomolgus macaques (Macaca fascicularis). Zool Res. 2019;40:89–93. 30127329 10.24272/j.issn.2095-8137.2018.061 PMC6378564

[51] Groves HT, Cuthbertson L, James P, Moffatt MF, Cox MJ, Tregoning JS. Respiratory disease following viral lung infection alters the murine gut microbiota. Front Immunol. 2018;9:182–193. 29483910 10.3389/fimmu.2018.00182 PMC5816042

[52] Carfì A, Bernabei R, Landi F. for the Gemelli against COVID-19 post-acute care study group. Persistent symptoms in patients after acute COVID-19. JAMA. 2020;324:603–605. 32644129 10.1001/jama.2020.12603 PMC7349096

[53] Muñoz-Fontela C, Dowling WE, Funnell SGP, Gsell P-S, Riveros-Balta AX, Albrecht RA, Andersen H, Baric RS, Carroll MW, Cavaleri M, et al. Animal models for COVID-19. Nature. 2020;586:509–515. 32967005 10.1038/s41586-020-2787-6 PMC8136862

[54] Lescure F-X, Bouadma L, Nguyen D, Parisey M, Wicky P-H, Behillil S, Gaymard A, Bouscambert-Duchamp M, Donati F, Le Hingrat Q, et al. Clinical and virological data of the first cases of COVID-19 in Europe: a case series. Lancet Infect Dis. 2020;20:697–706. 32224310 10.1016/S1473-3099(20)30200-0 PMC7156120

[55] Pizzorno A, Padey B, Julien T, Trouillet-Assant S, Traversier A, Errazuriz-Cerda E, Fouret J, Dubois J, Gaymard A, Lescure F-X, et al. Characterization and treatment of SARS-CoV-2 in nasal and bronchial human airway epithelia. Cell Rep Med. 2020;1:100059. 32835306 10.1016/j.xcrm.2020.100059 PMC7373044

[56] Lamas B, Richard ML, Leducq V, Pham H-P, Michel M-L, Da Costa G, Bridonneau C, Jegou S, Hoffmann TW, Natividad JM, et al. CARD9 impacts colitis by altering gut microbiota metabolism of tryptophan into aryl hydrocarbon receptor ligands. Nat Med. 2016;22:598–605. 27158904 10.1038/nm.4102 PMC5087285

[57] Bolyen E, Rideout JR, Dillon MR, Bokulich NA, Abnet CC, Al-Ghalith GA, Alexander H, Alm EJ, Arumugam M, Asnicar F, et al. Reproducible, interactive, scalable and extensible microbiome data science using QIIME 2. Nat Biotechnol. 2019;37:852–857. 31341288 10.1038/s41587-019-0209-9 PMC7015180

[58] Quast C, Pruesse E, Yilmaz P, Gerken J, Schweer T, Yarza P, Peplies J, Glöckner FO. The SILVA ribosomal RNA gene database project: improved data processing and web-based tools. Nucleic Acids Res. 2012;41:D590–6. 23193283 10.1093/nar/gks1219 PMC3531112

[59] McMurdie PJ, Holmes S. phyloseq: an R package for reproducible interactive analysis and graphics of microbiome census data. PLoS ONE. 2013;8:e61217. 23630581 10.1371/journal.pone.0061217 PMC3632530

[60] Segata N, Izard J, Waldron L, Gevers D, Miropolsky L, Garrett WS, Huttenhower C. Metagenomic biomarker discovery and explanation. Genome Biol. 2011;12:R60. 21702898 10.1186/gb-2011-12-6-r60 PMC3218848

[61] Lefèvre A, Mavel S, Nadal-Desbarats L, Galineau L, Attucci S, Dufour D, Sokol H, Emond P. Validation of a global quantitative analysis methodology of tryptophan metabolites in mice using LC-MS. Talanta. 2019;195:593–598. 30625588 10.1016/j.talanta.2018.11.094

[62] Humbert L, Maubert MA, Wolf C, Duboc H, Mahé M, Farabos D, Seksik P, Mallet JM, Trugnan G, Masliah J, et al. Bile acid profiling in human biological samples: comparison of extraction procedures and application to normal and cholestatic patients. J Chromatogr B. 2012;899:135–145. 10.1016/j.jchromb.2012.05.015 22664055

[63] Zheng X, Qiu Y, Zhong W, Baxter S, Su M, Li Q, Xie G, Ore BM, Qiao S, Spencer MD, et al. A targeted metabolomic protocol for short-chain fatty acids and branched-chain amino acids. Metabolomics. 2013;9:818–827. 23997757 10.1007/s11306-013-0500-6 PMC3756605

