# Supplementary figures and images for: SARS-CoV-2 infection in nonhuman primates alters the composition and functional activity of the gut microbiota
Source: Gut Microbes. 2021 Mar 8;13(1):1893113. doi: 10.1080/19490976.2021.1893113 (PMC7951961; doi:10.1080/19490976.2021.1893113)

**A**

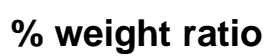

# B

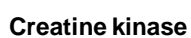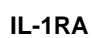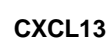

Supplement: Supplemental Material [file KGMI_A_1893113_SM9414.zip › supplemental Figure 1 revised.pdf]

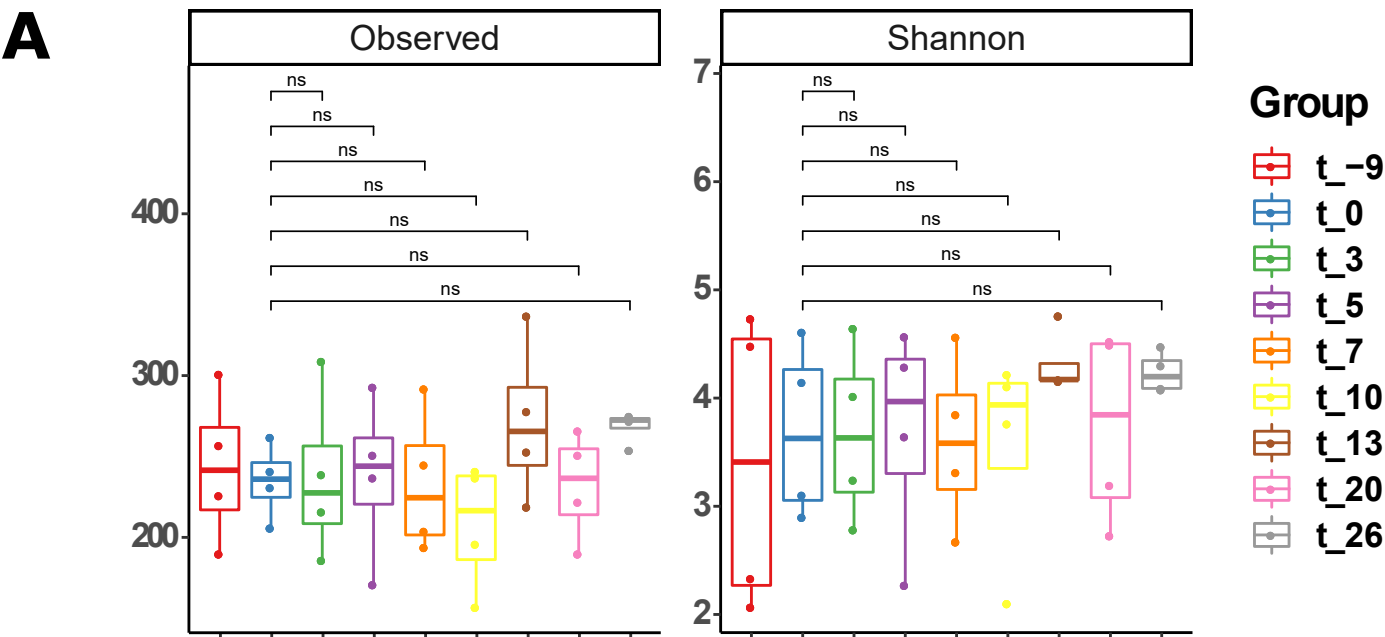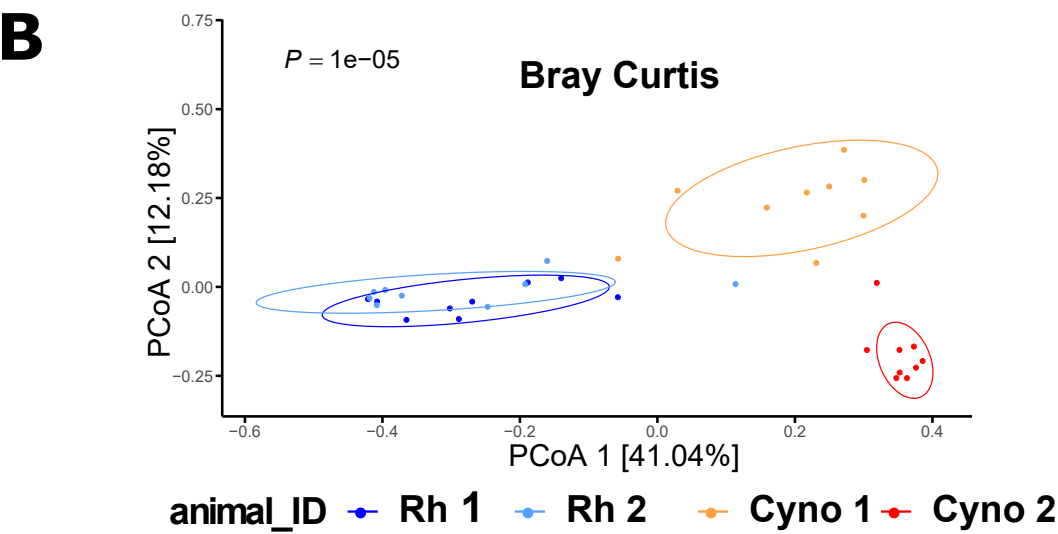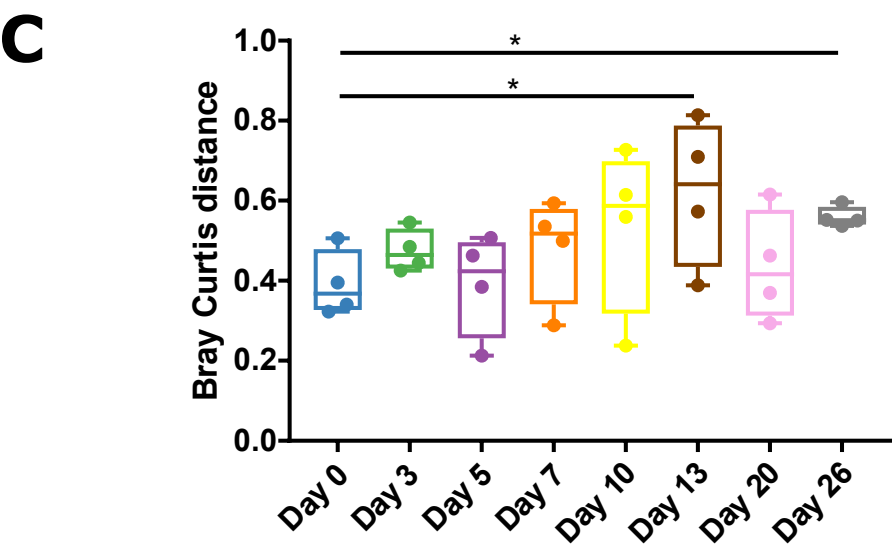

Supplement: Supplemental Material [file KGMI_A_1893113_SM9414.zip › Supplemental Figure 2 revised.pdf]

**A**

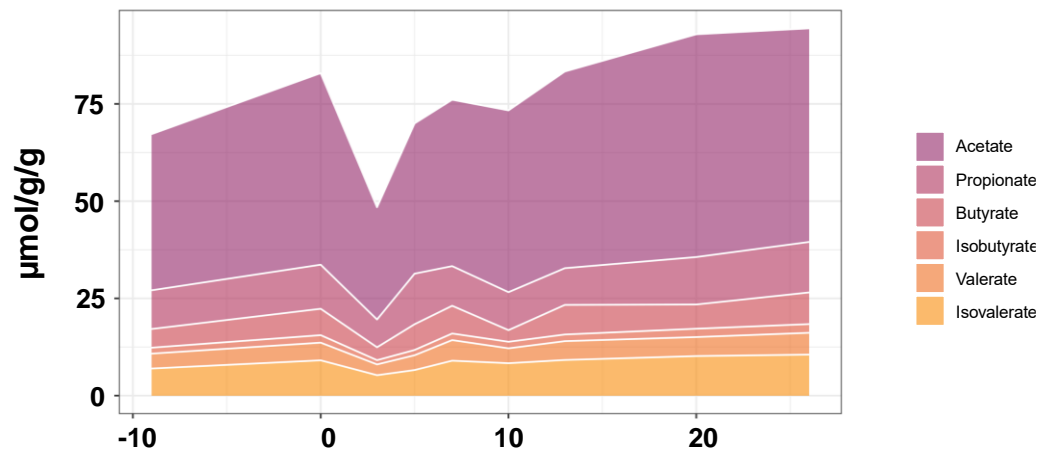

**B**

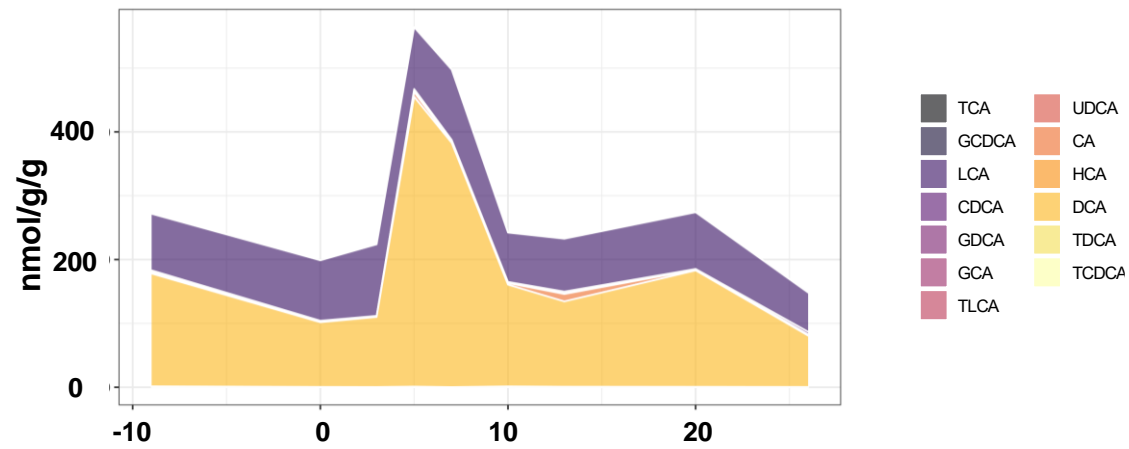

**C**

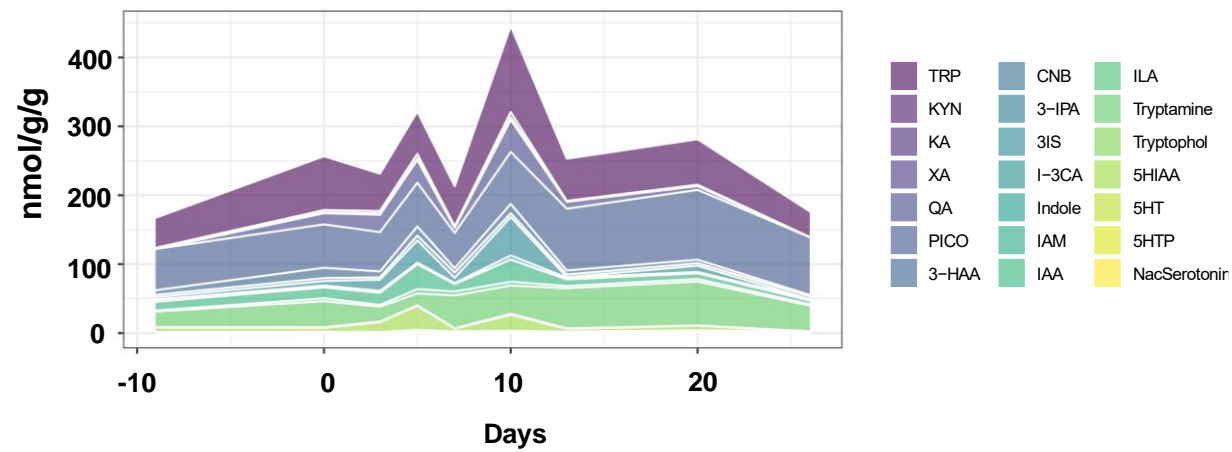

Supplement: Supplemental Material [file KGMI_A_1893113_SM9414.zip › supplemental Figure 3 revised.pdf]

# A

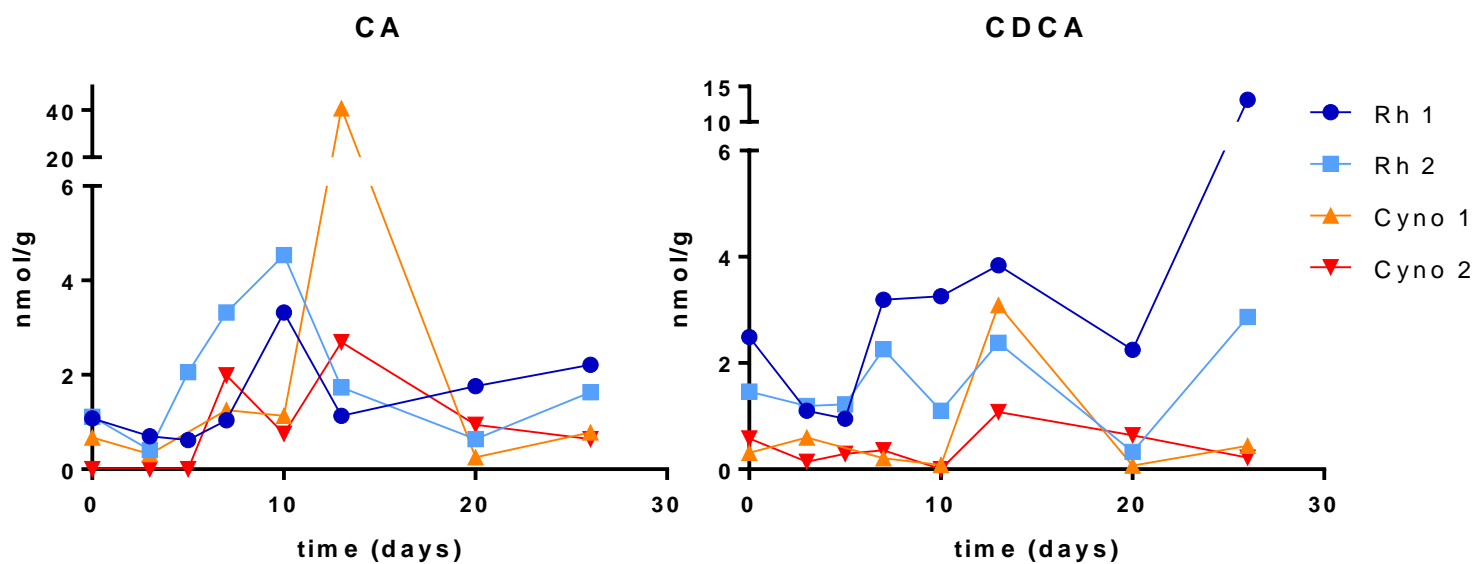

# B

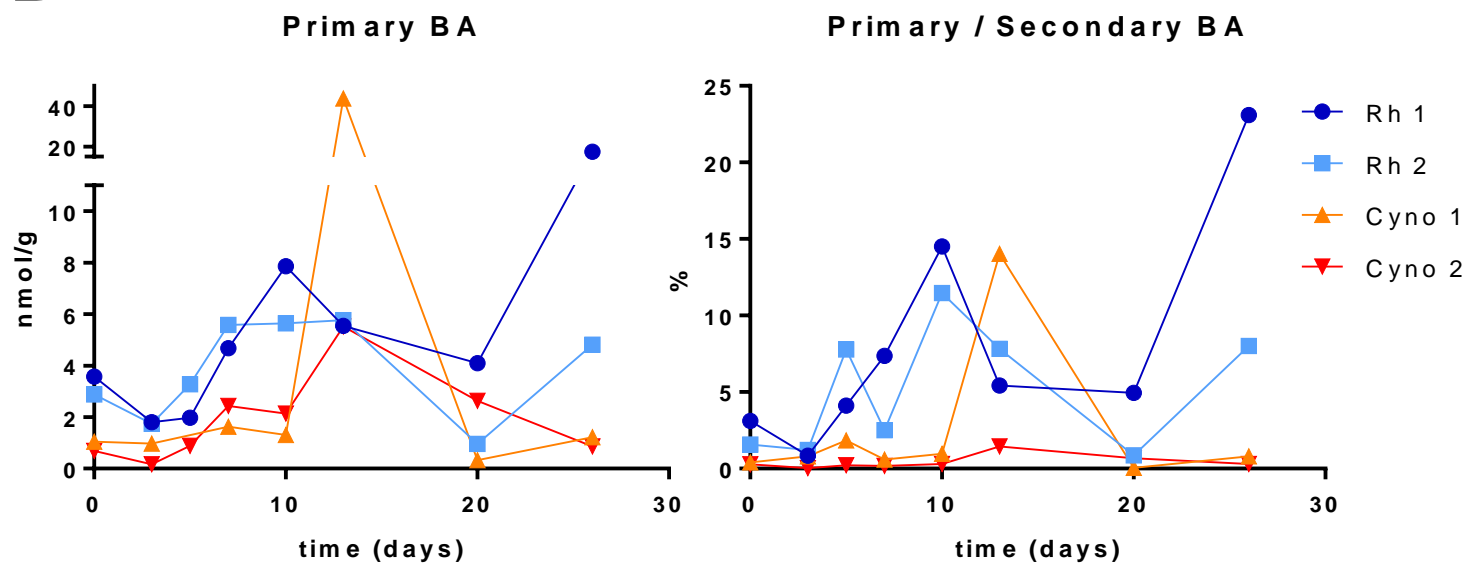

Supplement: Supplemental Material [file KGMI_A_1893113_SM9414.zip › supplemental Figure 4 revised.pdf]

**A**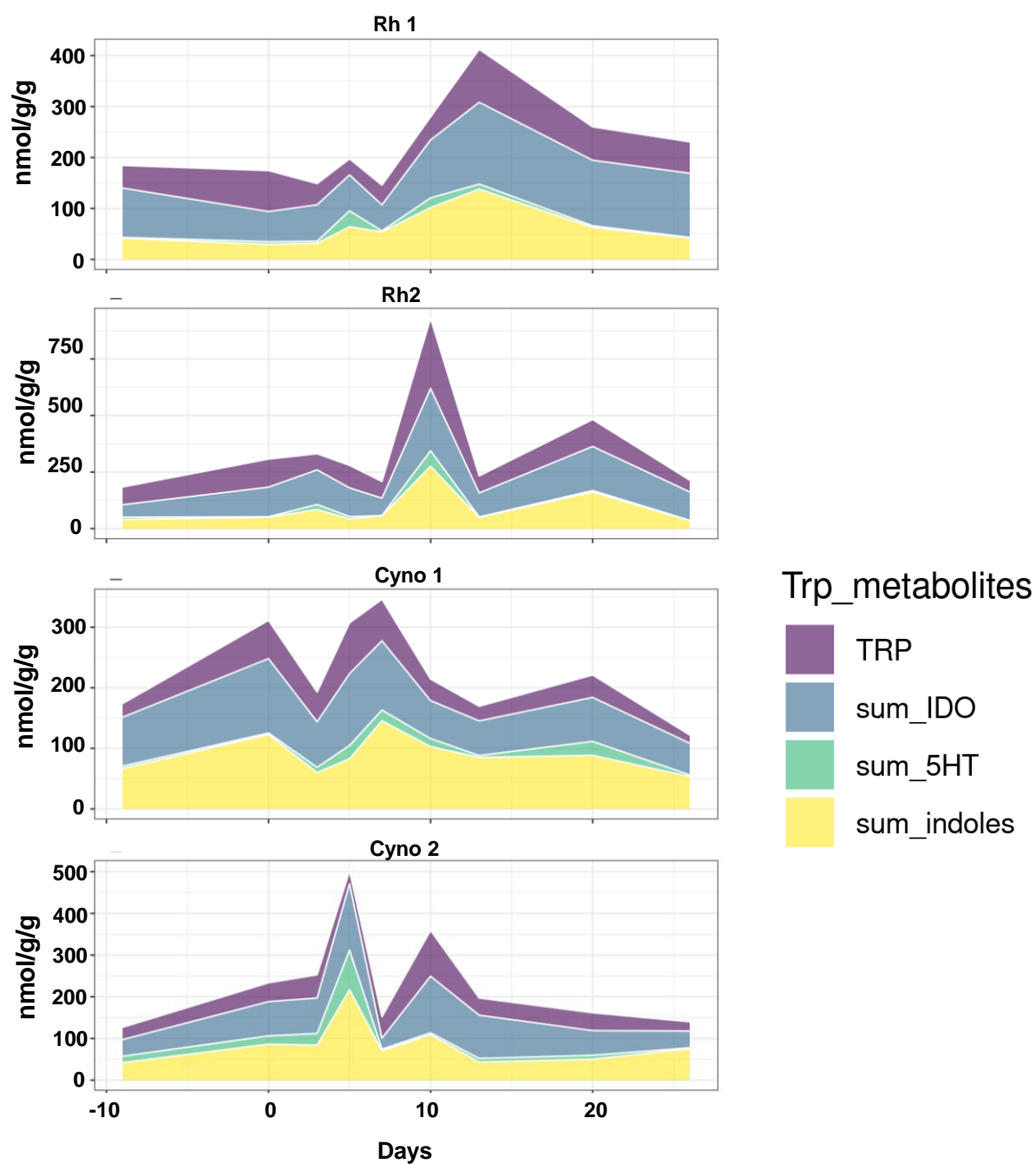**B**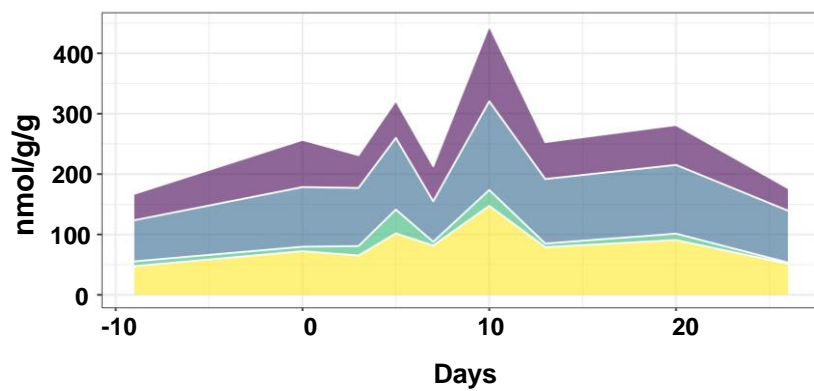

Supplement: Supplemental Material [file KGMI_A_1893113_SM9414.zip › supplemental Figure 5 revised.pdf]
